# Epidemiology of Candidemia, Candiduria and Emerging *Candidozyma* (*Candida*) *auris* Across Gulf Cooperative Council Countries and Yemen in the Arabian Peninsula

**DOI:** 10.1111/myc.70073

**Published:** 2025-06-09

**Authors:** Suhail Ahmad, Teun Boekhout, Abdullah M. S. Al‐Hatmi, Ahmed Al‐Harrasi, Aiah Mustafa Khateb, Fatima Al Dhaheri, Hajer Bin Shuraym, Jens Thomsen, Khaled Alobaid, Mohammad Asadzadeh, Saad J. Taj‐Aldeen, Saleh Alwasel, Wadha Alfouzan, Ziauddin Khan, Husam Salah

**Affiliations:** ^1^ Department of Microbiology, Faculty of Medicine Kuwait University Jabriya Kuwait; ^2^ Department of Zoology College of Sciences, King Saud University Riyadh Saudi Arabia; ^3^ The Yeasts Foundation Amsterdam the Netherlands; ^4^ Natural & Medical Sciences Research Centre University of Nizwa Nizwa Oman; ^5^ Department of Clinical Laboratory Sciences Collage of Applied Medical Science, Taibah University Medina Saudi Arabia; ^6^ Special Infectious Agents Unit, King Fahd Medical Research Center King Abdulaziz University Jeddah Saudi Arabia; ^7^ Department of Pediatrics College of Medicine and Health Sciences, United Arab Emirates University Al Ain UAE; ^8^ Department of Clinical Laboratory Sciences College of Applied Medical Sciences, King Saud Bin Abdulaziz University for Health Sciences Riyadh Saudi Arabia; ^9^ King Abdullah International Medical Research Center Riyadh Saudi Arabia; ^10^ Department of Public Health and Epidemiology Khalifa University Abu Dhabi UAE; ^11^ Medics Labor AG Bern Switzerland; ^12^ Microbiology Department, Mycology reference laboratory Mubarak Al‐Kabeer Hospital Jabriya Kuwait; ^13^ Division of Microbiology, Department of Laboratory Medicine and Pathology Hamad Medical Corporation Doha Qatar; ^14^ Microbiology Unit, Department of Laboratories Farwaniya Hospital Farwaniya Kuwait

**Keywords:** Arabian peninsula, *Candida*, epidemiology, gulf cooperation council countries, infections, yeasts

## Abstract

*Candida* infections represent a major component of invasive and non‐invasive mycoses globally, including the countries in the Arabian Peninsula. In this review, we present epidemiological features and trends, clinical manifestations, species distribution, antifungal susceptibility, and outcomes available for candidemia and candiduria in six countries of the Gulf Cooperation Council (GCC) and Yemen, all located in the Arabian Peninsula. We discuss gaps in knowledge and provide recommendations for improving various aspects for better management of infections by these fungal pathogens. *Candida* species prevail, with *Candida albicans* being the most isolated organism, though its prevalence varies over time. The second most frequently isolated species varies from country to country within the region. Generally, invasive infections by non‐*albicans Candida* species are increasing. *Candidozyma auris,* formerly known as *Candida auris,* is causing serious health risks in all GCC countries, including those with appropriate diagnostic capacity and awareness.

## 1 Introduction

Yeast infections represent a major component of invasive and non‐invasive mycoses globally [1, 2, 3, 4, 5]. During the last few decades, the incidence of fungal infections has steadily increased, particularly in immunocompromised patient populations [1, 2, 6, 7]. Additionally, the emergence of drug‐resistant fungi poses new therapeutic challenges [8, 9]. Intense use of cytotoxic drugs for the treatment of cancers, the availability of novel immunosuppressive formulations for autoimmune disorders, the increasing acceptance of surgical procedures for cosmetic purposes, the use of modern facilities for organ and bone marrow transplantation, and extended survival of debilitated patients in intensive care units (ICUs) are some of the factors contributing to the rising incidence of fungal infections [10, 11, 12]. *Candida* colonisation is regarded as one of the predisposing risk factors for invasive infections, and recent studies have shown that *Candida* species can easily translocate from various anatomic sites to the bloodstream, causing candidemia and invasive candidiasis (IC) [3, 13, 14, 15]. Previous epidemiological studies have shown that > 90% of invasive yeast infections are caused by only five species/species complexes that include *Candida albicans* , *Nakaseomyces glabratus* (formerly known as *Candida glabrata*), *Candida parapsilosis sensu lato*, *Candida tropicalis* , and *Pichia kudriavzevii* (formerly known as *Candida krusei* ) [11, 12, 16]. A gradual shift to non‐*albicans Candida* (NAC) species has been recently noted in various regions [2, 17, 18, 19].

The World Health Organization (WHO) has recently released a fungal priority pathogen list (WHO FPPL) [20], with fungal pathogens being classified into three (critical, high, and medium) groups. Among these, several yeast pathogens, namely *C. albicans, Candidozyma auris* (*Cz. auris*, formerly known as *Candida auris* [21]), and *Cryptococcus neoformans* species complex, are part of the critical group. *N. glabratus, C. tropicalis*, and *C. parapsilosis sensu stricto* are placed in the high‐priority group, while *P. kudriavzevii* and the *Cryptococcus gattii* species complex are pathogens in the medium category [20]. Among critically ill and immunocompromised patients, invasive fungal infections (IFI), which include IC and candidemia, are serious health concerns worldwide. There is data scarcity in the Middle East, especially from countries in the Arabian Peninsula, which include Bahrain, Kuwait, Qatar, Oman, Saudi Arabia, and the United Arab Emirates (UAE), together with Yemen. GCC countries have limited or no national monitoring systems for fungal diseases, further exacerbating the knowledge gap in the region. Additionally, susceptibility testing is not uniformly done in many of these countries [22, 23]. The overall incidence of IC varied throughout the region, with Kuwait having 0.15 cases per 1000 hospital discharges and Saudi Arabia having 1.55–1.65 cases per 1000 hospital discharges [22]. Recent data from these countries in the last decade revealed that *C. albicans* was the most frequently isolated yeast species in Kuwait (37.2%–47.2%) [2, 24], Qatar (30.2%) [25], and UAE (45%) [26]. The emergence of the multidrug‐resistant *Cz*. *auris* has become a global concern, including in the Middle East region. This species usually exhibits reduced susceptibility to azoles and amphotericin B [9, 27]. The United States Centers for Disease Control and Prevention (CDC) has recently declared *Cz. auris* as an urgent threat to public health [28]. Numerous studies and case reports on *Cz. auris* have been published from the GCC countries outlined below.

The taxonomy of fungi, including yeasts, is developing rapidly due to the application of refined molecular phylogenies that are increasingly based on whole genome data, but also the introduction of the nomenclatural principle of “One fungus = One name.” Consequently, many yeast species have been renamed according to their phylogenetic placement, which has resulted in name changes that initially may hamper diagnostics [21, 29, 30, 31]. Nevertheless, opposing views occur with respect to the need to introduce such name changes in the clinical setting [32, 33]. For the sake of clarity, a list of nomenclature changes in clinically relevant yeasts is presented in Table 1. Reference books such as The Yeasts, a Taxonomic Study [29] and continuously updated databases, such as theyeasts.org (https://theyeasts.org/) and mycobank.org (https://www.mycobank.org/), can be consulted for names of yeasts in current and past use.

**TABLE 1:** List of nomenclature changes in clinically relevant yeasts^a^.

New name	Old name
*Apiotrichum loubieri*	*Trichosporon loubieri*
*Apiotrichum mycotoxinivorans*	*Trichosporon mycotoxinivorans*
*Candidozyma auris*	*Candida auris*
*Candidozyma duobushaemuli*	*Candida duobushaemulonii*
*Candididozyma haemuli*	*Candida haemulonii*
*Candidozyma khanbhai*	*Candida khanbhai*
*Clavispora lusitaniae*	*Candida lusitaniae*
*Cryptococcus bacillisporus*	*Cryptococcus gattii* genotype VGIII, AFLP5
*Cryptococcus deneoformans*	*Cryptococcus neoformans* serotype D, *Filobasidiella neoformans*, *Cryptococcus neoformans* genotype VNIV, AFLP2
*Cryptococcus decagattii*	*Cryptococcus gattii* genotype VGII, AFLP6
*Cryptococcus deuterogattii*	*Cryptococcus gattii* genotype VGIIIc/IV, AFLP10
*Cryptococcus gattii*	*Filobasidiella gattii*, *Cryptococcus gattii* genotype VGI, AFLP4
*Cryptococcus neoformans*	*Cryptococcus neoformans* serotype A, *Filobasidiella neoformans*, *Cryptococcus neoformans* var. *grubii*, *Cryptococcus neoformans* genotype VNI, VNII, AFLP1, 1A, 1B
*Cryptococcus tetragattii*	*Cryptococcus gattii* genotype IV, AFLP7
*Cutaneotrichosporon dermatis*	*Trichosporon dermatis*
*Cutaneotrichosporon mucoides*	*Trichosporon mucoides*
*Cyberlindnera fabianii*	*Candida fabianii*
*Cyberlindnera jadinii*	*Candida utilis*
*Cystobasidium minutum*	*Rhodotorula minuta*
*Debaryomyces hansenii*	*Candida famata*
*Geotrichum candidum*	*Galactomyces candidus*
*Diutina rugosa*	*Candida rugosa*
*Filobasidium chernovii*	*Cryptococcus chernovii*
*Filobasidium magnum*	*Cryptococcus magnus*
*Filobasidium uniguttulatum*	*Cryptococcus uniguttulatus*
*Kluyveromyces marxianus*	*Candida kefyr*
*Kodamaea ohmeri*	*Pichia ohmeri*
*Lachancea fermentati*	*Candida fermentati*
*Magnusiomyces capitatus*	*Saprochaete capitata, Geotrichum capitatum, Dipodascus capitatus*
*Saprochaete clavata*	*Geotrichum clavatum*
*Meyerozyma caribbica*	*Candida fermentati*
*Meyerozyma guilliermondii*	*Candida guilliermondii*
*Naganishia adeliensis*	*Cryptococcus adeliensis*
*Naganishia albida*	*Cryptococcus albidus*
*Naganishia liquefaciens*	*Cryptococcus liquefaciens*
*Nakaseomyces bracarensis*	*Candida bracarensis*
*Nakaseomyces glabratus*	*Candida glabrata*
*Nakaseomyces nivariensis*	*Candida nivariensis*
*Papiliotrema laurentii*	*Cryptococcus laurentii*
*Pichia fermentans*	*Candida lambica*
*Pichia inconspicua*	*Candida inconspicua*
*Pichia kudriavzevii*	*Candida krusei*
*Pichia norvegensis*	*Candida norvegensis*
*Pichia ohmeri*	*Candida guilliermondii* var. *membranifaciens*
*Rhodotorula mucilaginosa*	*Rhodotorula rubra*
*Saccharomyces cerevisiae*	*Candida robusta*
*Wickerhamomyces anomalus*	*Candida pelliculosa*
*Yarrowia lipolytica*	*Candida lipolytica*

^a^ Based on Kurtzman et al. [29], Hagen et al. [30], Zhu et al. [31], Liu et al. [21], and The Yeasts Trust Database (www.https://theyeasts.org).

This review is limited to GCC countries and Yemen located at the Arabian Peninsula. The climate of this region is characterised by high temperatures in the summer (up to or > 50°C) and very limited precipitation (c. 75–00 mm per year), resulting in an arid climate with many deserts and desert‐like landscapes. Next to deserts, mountains, plateaus, inland seas, and high‐salt‐containing lakes or sabhka (Arabic for areas of low‐lying salty ground), wadi's (Arabic for dry river valleys with intermittent water flow during the rainy season), and a coastline do occur. The coastline can either be rocky or flat and may be covered with mangroves. Most of the approximately 80 million people living in the region live in cities, with several cities having populations > 1 million inhabitants. The local population mainly consists of Arabs, but also many expatriates from Western, Asian, and African countries live in this region [34, 35].

This review aims to comprehensively examine existing data on the epidemiology of candidemia and candiduria and the emerging *Cz. auris* from these countries, highlighting key trends, diagnostic challenges, and gaps in knowledge. By providing a detailed analysis of regional findings, this review intends to shed light on critical issues in *Candida* infection diagnosis, management, and prevention across the region.

We present a narrative review based on literature on the epidemiology of candidemia, candiduria, and the emerging *Cz. auris* across Gulf Cooperation Council (GCC) countries and Yemen. Given the relatively limited number of studies in the region and the heterogeneity in methodologies, we believe that a narrative review format is the most appropriate approach to comprehensively summarise findings and highlight diagnostic challenges and knowledge gaps. Country‐specific data were collected by co‐authors representing each respective country, except Yemen and Bahrain. Where available, unpublished national reports and surveillance summaries were also included to ensure comprehensive regional coverage. A literature search of articles in the English language was conducted using PubMed with combinations of the following keywords: *Candida*, candidemia, candiduria, fungemia, *Candida auris*, *Candidozyma auris*, invasive *Candida* infections, and invasive yeast infections, along with the names of each country. For countries where data were limited or unavailable in PubMed, such as Yemen, Google searches using the same keywords were used to identify additional relevant literature. Studies were included if they specifically reported data on candidemia, candiduria, or *Cz. auris*. Articles were excluded if they were not in the English language, addressed other types of *Candida* or yeast infections (e.g., superficial infections), or did not provide relevant epidemiological data.

## 2 Burden of Yeast‐Related Infections

**FIGURE 1:**
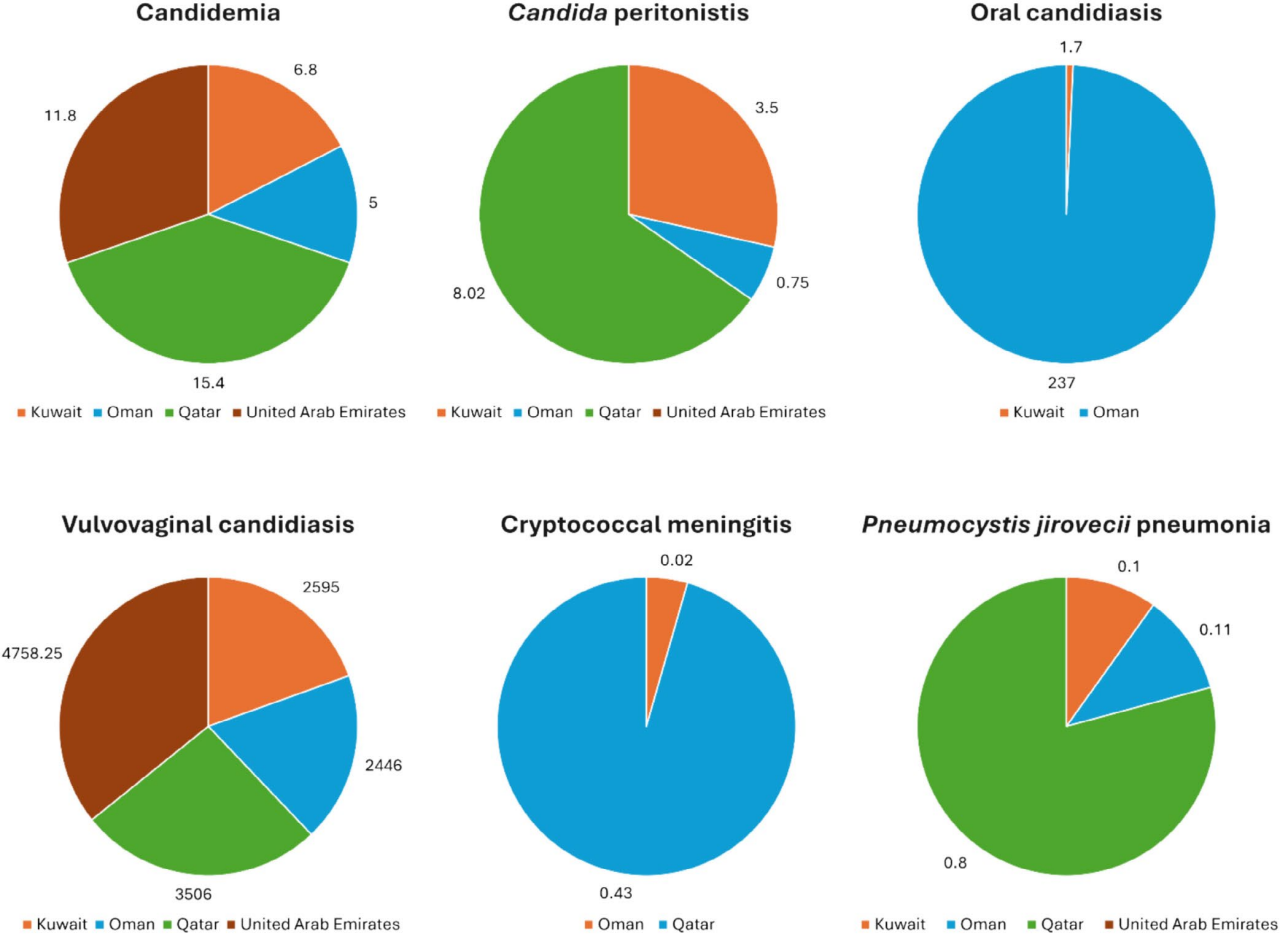
Incidence (number of cases/100,000 population) of yeast‐related infections from selected countries in the Arabian Peninsula^a^. ^a^Data are based on Alobaid et al. [4], Alfouzan et al. [36], Al‐Hatmi et al. [37], Taj‐Aldeen et al. [38], Al Dhaheri et al. [39].

Data on the incidence of yeast‐related infections were reported from Kuwait, Oman, Qatar, and the UAE, but not from Bahrain, Saudi Arabia, and Yemen. Figure 1 highlights the reported burden of the common *Candida* infections in the region. Table 2 provides an overview of the affected populations, risk factors, and predominant yeast species observed in these infections across the region.

**TABLE 2:** Epidemiology of yeast‐related infections in GCC countries and Yemen: Affected populations, risk factors, and predominant species.

Country	Type of infection	Risk factors	Mortality (%)	Common species	References
Bahrain	Candidemia	Neonatal sepsis, COVID‐19, HIV	70.4	*Candida albicans, Candida tropicalis, Nakaseomyces glabratus, Candida parapsilosis*	[40, 41, 42]
	Candiduria	High SAPSII score, CVC, mechanical ventilation, renal replacement therapy, prior antibiotic therapy	62.1	*C. albicans, C. tropicalis, N. glabratus, C. parapsilosis, Kluyveromyces marxianus, Clavispora lusitaniae*	[43]
Kuwait	Candidemia	Broad‐spectrum antibiotics, steroids, TPN, CVC, hospitalisation, neonates, immunosuppression, ICU admission, adults > 65 years, diabetes, cancer, abdominal surgery, use of vascular catheters, haemodialysis, antibiotic exposure	29.5–54	*C. albicans, C. tropicalis, N. glabratus, C. parapsilosis, K. marxianus, C. lusitaniae, Candidozyma haemuli, Debaryomyces hansenii, Candida humicola, Candida lipolytica, Candida orthopsilosis, Candida metapsilosis, Pichia kudriavzevii, Candida dubliniensis, N. glabratus, Candidozyma auris, Lodderomyces elongisporus, Kodamaea ohmeri, Cyberlindnera fabianii, Candida blankii, Meyerozyma guilliermondii, Candida pelliculosa*	[2, 4, 24, 44, 45, 46]
	Candiduria	Urinary catheter, urinary tract infection, kidney disease, on broad‐spectrum antibiotics, diabetes, cancer, abdominal surgery, gastrointestinal bleeding, liver disease, candidemia, septic shock, respiratory infection/failure, brain injury, adults > 65 years, prematurity, congenital nephrotic syndrome, AML, kidney transplant, burn, chronic kidney disease, steroids and rituximab treatment	NA	*C. tropicalis, C. lusitaniae, C. parapsilosis, C. orthopsilosis, N. glabratus, C. albicans, K. marxianus*	[47, 48, 49, 50, 51, 52, 53, 54, 55, 56]
Oman	Candidemia	Leukaemia, CVC, broad‐spectrum antibiotics, COVID‐19, ICU admission, Burn, Cancer	40.7–60	*C. tropicalis, C. parapsilosis, C. albicans, N. glabratus, Candida* spp., *P. kudriavzevii, C. dubliniensis, K. marxianus*	[57, 58, 59, 60, 61]
	Candiduria	NA	NA	*Candida* spp.	[56]
Qatar	Candidemia	Neonates, elderly, heart/pulmonary disorders, haematological malignancies, solid organ tumours, abdominal surgery, renal diseases, renal transplantation, ICU admission, surgery, cancer/malignancies, age, assessment score, immunosuppressive therapy	56.1–60	*C. albicans, C. tropicalis, C. parapsilosis, C. orthopsilosis, N. glabratus, K. marxianus, Kluyveromyces lactis, L. elongisporus, C. dubliniensis, M. guilliermondii, P. kudriavzevii, Yarrowia lipolytica, Candida rugosa, Candida pararugosa, Candida intermedia, Cy. fabianii, Wickerhamomyces anomalus, Candida* spp., *Candida bracarensis, Cz. auris, D. hansenii, K. ohmeri*	[25, 62, 63]
	Candiduria	Diabetes, refractory urine retention, transurethral resection of the prostate	NA	*C. albicans*	[64]
Saudi Arabia	Candidemia	Cancer, pre‐term infants, surgery, CVC, broad‐spectrum antibiotics, abdominal surgeries, TPN, neutropenia, acute renal failure, malignancy, burns, ICU stay, urinary catheters, immunosuppressive chemotherapy, intra‐abdominal infection, low birth weight, ventilator support, immunotherapy, renal diseases, GIT diseases, respiratory diseases, cardiovascular diseases, trauma, age, previous antifungal therapy, hyperalimentation, diabetes, organ transplantation	40.6–71	*C. albicans, C. tropicalis, C. parapsilosis, N. glabratus, P. kudriavzevii, D. hansenii, C. dubliniensis, C. lusitaniae, M. guilliermondii, C. haemuli, Candida spherica*	[65, 66, 67, 68, 69, 70, 71, 72, 73, 74]
	Candiduria	NA	NA	*C. albicans, C. tropicalis, C. parapsilosis, N. glabratus*	[73]
United Arab Emirates	Candidemia	Haematological malignancy/lymphoma, solid tumours, antibiotic therapy, neutropenia, bacteremia, old age, ICU admission, renal failure	50	*C. albicans, C. tropicalis, N. glabratus, C. parapsilosis, Candida inconspicua, D. hansenii, C. lusitaniae, Cz. auris*	[26, 75]
	Candiduria	NA	NA	NA	—
Yemen	Candidemia	NA	NA	NA	—
	Candiduria	Urinary catheters, Diabetes, antibiotic therapy		*C. albicans* , NAC species.	[76]

Abbreviations: AML, acute myeloid leukaemia; CVC, central venous catheter; GIT, gastrointestinal tract; ICU, intensive care unit; NA, data not available; NAC, non‐*albicans* *Candida*; SAPSII, Simplified Acute Physiology Score II; TPN, total parenteral nutrition.

### 2.1 Bahrain

Data on the burden of yeast‐related infections have not been reported from Bahrain. Only a few reports, outlined below, have documented cases of candidemia, candiduria, and *Cz. auris* infection/colonisation.

### 2.2 Kuwait

Extensive data exist on the extent of yeast infections in this country. The annual occurrence of fungal infections in Kuwait has been calculated for the year 2018. The absolute number of cases reported (and their incidence, cases per 100,000 in parenthesis) included recurrent *Candida* vaginitis, 54,842 (2595), candidemia 234 (5.29) [4], *Candida* peritonitis 63 (3.5), and oesophageal candidiasis in HIV 33 (0.8) [36].

### 2.3 Oman

Fungal infections, caused by both yeasts and moulds, have received little attention, and no comprehensive literature exists on fungal infections in Oman. A study by Al‐Hatmi et al. [37] estimated that 79,520 individuals in Oman suffer from a severe fungal infection annually, which equates to 1.7% of the country's population (excluding fungal skin infections, chronic fungal rhinosinusitis, and otitis externa). Estimates for Oman's epidemiological burden of yeast infections show that the country has the following incidence rates/100,000 patients: 5 for candidemia; 0.75 for *Candida* peritonitis; 2446 for recurrent *Candida* vaginitis; 237 for oral candidiasis; and 1.5 for oesophageal candidiasis. This estimate also accounts for a few rare yeast infections, such as cryptococcal meningitis (0.02/100,000) [37].

### 2.4 Qatar

Taj‐Aldeen et al. [38] evaluated the burden of fungal infections in Qatar over a 5‐year period (2009–2014). These data were collected from the Microbiology Laboratory database of Hamad Medical Corporation in Doha as well as from 2011 WHO statistics. Cases of candidemia rose to 288 at an approximate rate of 15.4/100,000, which was higher than that reported for the period 2004–2009 (12.9/100,000). *Candida* peritonitis was estimated to occur in 8.02/100,000 individuals. Recurrent vaginal infections were reported for at least 32,782 women, with a rate of 3506/100,000 inhabitants. The low rate of *Cryptococcus* meningitis (0.43/100,000) was likely due to the low prevalence of HIV infection in the country [38] and similar to other GCC countries [77].

### 2.5 Saudi Arabia

Invasive yeast infections, particularly those caused by species of *Candida*, present a significant concern for public health in Saudi Arabia. *C. albicans* is the most common causative agent, but other species like *N. glabratus* and *C. parapsilosis* are also common [6]. Despite several studies on yeast infections, a comprehensive report on the overall burden in Saudi Arabia has not been published. The precise incidence varied across regions and patient populations, but studies suggest rates ranging from 0.45 to 1.65 cases/1000 hospital discharges [6, 65]. Candidemia accounts for around 4% of positive blood cultures in some hospitals, making it a major cause of hospital‐acquired bloodstream infections [78]. Another study looked specifically at the annual incidence of candidemia in Saudi Arabia and found that it was fluctuating between 0.2 and 0.76 cases/1000 discharges, with variations observed in different regions of the country [79].

### 2.6 United Arab Emirates (UAE)

The data on the incidence and prevalence of yeast infections in the UAE are limited. Recently, an estimate of the burden of fungal infections in the country was reported by Al Dhaheri et al. [39]. A total of 190,330 cases of recurrent vulvovaginal candidiasis (RVVC), two cases of cryptococcal meningitis, and 20 HIV‐related oesophageal candidiasis cases were reported. A statistically significant shift was observed during 2010–2020. *C. albicans* was the major yeast pathogen in 2010, accounting for 78.6% of all *Candida* spp. Isolates, while this value declined to 47.0% in 2020. Conversely, the proportion of NAC species among all *Candida* spp. increased from 21.4% (2010) to 53.8% (2020). This increase of NAC species over time was likely caused by an increased reporting of *C. tropicalis, C. parapsilosis*, and *Cz. auris* [19].

### 2.7 Yemen

Not much has been published regarding the incidence of yeast infections in Yemen. Most of the available reports (addressed below) involved oral candidiasis and RVVC.

## 3 Candidemia

### 3.1 Bahrain

In a long‐term (1991–2001) study on the microbial diversity involved in neonatal sepsis from Bahrain, 335 of 7978 (4.2%) neonates had culture proven candidemia. Although detailed analysis of yeast species was not presented, the authors noted an increasing trend in the detection of *Candida* (mainly *C. albicans* ) from 2.4% to 6.5% [40]. Similar findings were also reported in a study on neonatal sepsis involving 12 hospitals in Bahrain and Kuwait [80]. In an earlier study (May 1997 to November 1998) involving HIV‐infected individuals with low CD4+ T‐lymphocyte counts, *C. albicans* was the cause of infection in 11 of 21 (52.4%) patients [41]. Although data on the source of isolation for *C. albicans* was not available, it is likely that at least some of these cases were bloodstream infections. A more recent retrospective observational study by Saeed et al. [42] has reported that candidemia is the second most common nosocomial infection after bacterial infections among 30 patients with COVID‐19. The species involved included *N. glabratus* (*n* = 11), *C. tropicalis* (*n* = 9), *C. albicans* (*n* = 7), and *C. parapsilosis* (*n* = 3).

### 3.2 Kuwait

The first candidemia study in Kuwait was published in 2002 and showed that *C. albicans* was the dominant species, followed by *C. parapsilosis*, while *C. tropicalis* and *N. glabratus* were the least frequently isolated species [81].

The first comprehensive study on 607 candidemia patients analysed bloodstream *Candida* isolates collected over a 10‐year period (1996–2005). The study showed that infections with *C. albicans* were more common (39.5%) than those caused by *C. parapsilosis* (30.6%) and *C. tropicalis* (12.4%). *N. glabratus* (5.6%) and *P. kudriavzevii* (1.6%) were less commonly isolated, while 10.2% of cases were caused by other *Candida* or yeast species [24]. In another study, *Candida* spp. isolates were identified among neonatal candidemia patients (*n* = 153) over a 12‐year period (1995–2006). Here, *C. parapsilosis* was detected as the dominant species (49%) rather than *C. albicans* (41.2%) [44].

There has been a significant epidemiological shift in the relative prevalence of *C. albicans* and *C. parapsilosis* among candidemia patients after 2005. A subsequent comprehensive study evaluated the impact of antifungal therapy on the incidence and susceptibility profiles of the six common *Candida* bloodstream isolates (*n* = 2075) collected over a 12‐year period. The data were split over two 6‐year periods: 2006–2011 (*n* = 970) and 2012–2017 (*n* = 1105) for comparative analyses [2, 82]. Although *C. albicans* (37.0%) was the dominant species during the first 6‐year period of this study, *C. parapsilosis sensu lato* (37.7%) became more common in the subsequent 6‐year period, followed by *C. tropicalis* (13.3%), *N. glabratus* (9.2%) and *P. kudriavzevii* (1.5%) and *Candida dubliniensis* (1.3%). There was also an increasing trend in candidemia cases caused by *Cz. auris* in the 2012–2017 period [2, 45].

The predominance of NAC species among total candidemia cases (*n* = 89) was also apparent in another study that was performed at a major secondary‐care (viz., Mubarak Al‐Kabeer) hospital in Kuwait during 2014–2016 as only 32% of cases were caused by *C. albicans*, while *C. parapsilosis sensu lato*, *C. tropicalis* , and *N. glabratus sensu lato* accounted for 65% of the cases. The remaining 3% cases were caused by rare *Candida* or other yeast species (i.e., one case each of *Cz. auris*, *Debaryomyces hansenii* , and *C. dubliniensis*) [46]. The 30‐day all‐cause mortality was 54% [46]. The impact of *Cz. auris* was more pronounced in 2018, as it surpassed the fourth‐ranked *N. glabratus*, and cases caused by *C. albicans* declined further to only 31%. Another noteworthy observation was the detection of resistance to fluconazole in 9 of 54 (16.7%) *C. parapsilosis* sensu stricto isolates in 2018 [4].

### 3.3 Oman

A study on the occurrence of IFI in children with leukaemia (*n* = 198) below 13 years of age between 2010 and 2017 found that 28% of paediatric patients with IFI developed candidemia. *C. tropicalis* (15.6%, *n* = 32) was the most common organism, followed by *C. parapsilosis* (6.3%, *n* = 32) [57]. El‐Hussain et al. (2022) reported that 227 of the 400 medical prescriptions were written for infections caused by *Candida* spp., which comprised 83.8% of the positive fungal cultures. Among the *Candida* positive cultures (*n* = 119), 15.1% were identified as *Cz. auris* [83]. Anidulafungin, the first‐line drug recommended for candidemia, was prescribed for 52.5% of patients (*n* = 421), suggesting that these individuals likely had candidemia [83]. Three cases of candidemia due to *C. albicans* , *N. glabratus* , and *C. tropicalis* were reported from adult patients with COVID‐19 [58] at a single centre.

A retrospective study involving all isolates recovered at the microbiology laboratory of Suhar Hospital between 2016 and 2017 showed that *C. albicans* was the most isolated species; however, the prevalence of NAC and non‐*Candida* yeast species was steadily increasing. Hospital‐acquired blood stream infections (HA‐BSIs) were found in 268 of 30,764 patients admitted to Sultan Qaboos University Hospital in Oman [84], implying a prevalence rate of 8.7 cases per 1000 admissions. Most HA‐BSI patients suffered from monomicrobial infections, and *Candida* spp. (764; 9.4%, *n* = 30,764) were the third most frequently identified pathogen [84].

A recent retrospective study (2013–2021) described the epidemiology, risk factors, and outcomes of chronic disseminated candidiasis in children at Sultan Qaboos University Hospital, Oman. Six from 36 (16.7%) immunocompromised children diagnosed with IFI had chronic disseminated candidiasis, and all were with acute leukaemia. Common clinical features included prolonged fever and skin rash. Immune reconstitution inflammatory syndrome (IRIS) associated with chronic disseminated candidiasis occurred in five children (83%), two of whom received corticosteroid therapy [59].

Over half (i.e., 50.9%) of major burn patients had positive blood cultures, with *Candida* species frequently isolated. These findings underscore the importance of considering fungal pathogens, particularly candidemia, in the management of bloodstream infections in burn patients, highlighting the need for appropriate antifungal therapy alongside antibacterial treatments [60].

Awada et al. (2024) recently investigated the epidemiology, antifungal resistance, and risk factors for mortality in solid tumour cancer patients with candidemia at the Sultan Qaboos Comprehensive Cancer and Research Centre. The study included adult patients admitted with candidemia over 2 years. Among 5469 admissions, 27 candidemia cases were identified, resulting in an incidence rate of 4.9 per 1000 admissions. *N. glabratus* (37%) was the most common species, followed by *C. albicans* (29.6%). Antifungal susceptibility testing revealed that most *Candida* species, including *C. albicans* and *C. tropicalis* , were susceptible to antifungal agents like fluconazole and echinocandins, but some resistance was noted in *N. glabratus* and *P. kudriavzevii*. The 30‐day crude mortality rate was 40.7%, with ICU admission being a significant risk factor for mortality. The study highlights the prevalence of NAC species and the importance of appropriate antifungal therapy in reducing candidemia‐related mortality in cancer patients [61].

### 3.4 Qatar

A 6‐year retrospective study evaluated the epidemiology, risk factors, demographic characteristics, species distribution, and clinical outcome linked with candidemia among patients admitted to Hamad General Hospital (HGH) in Qatar [62]. *Candida* isolates (*n* = 201) were identified using sequence‐based molecular methods and MALDI‐TOF MS. *C*. *albicans* was the predominant species isolated (33.8%), followed by various NAC species (66.2%). Patients with NAC species exhibited a higher mortality rate (68%–71.4%) in comparison to those infected with *C. albicans* . Moreover, the highest mortality rates among candidemia patients were observed among patients < 1 year‐old and above 60 years old. Taj‐Aldeen et al. (2018) [25] analysed 301 *Candida* bloodstream isolates over 5 years from a tertiary hospital in Qatar and reported that 53% of patients were admitted to the ICUs. NAC species steadily increased over the study period (30.2% for *C. albicans* compared to 69.8% for the other *Candida* species). Omrani et al. (2021) [63] investigated the incidence rate and risk factors for candidemia in patients with COVID‐19 in an ICU and reported an incidence rate of 2.34 per 1000 ICU days. The only risk factors independently associated with candidemia were age and sequential organ failure assessment score. On the other hand, administration of tocilizumab and corticosteroids was not independently associated with candidemia [63].

### 3.5 Saudi Arabia

In 2003, Al‐Hedaithy et al. showed that *C. albicans* and *C. dubliniensis* were responsible for 50.3% of the fungemia episodes (*n* = 189) in a Riyadh University hospital. Other species were also reported, such as *C. tropicalis* (27%), *C. parapsilosis* (7.9%), *N. glabratus* (7.4%), *P. kudriavzevii* (3.2%), and *Debaryomyces hansenii* (formerly known as *Candida famata* , 1%) [66]. A study from Eastern Saudi Arabia reported that during 1996–2004, *C. albicans* (53%) was the primary leading species causing IFI (*n* = 98), followed by *C. tropicalis* (19.4%), *C. parapsilosis* (16.3%), and *N. glabratus* (7.1%) [67]. More recent research showed that *C. albicans* accounted for 33% of candidemia cases (*n* = 326). Apparently, there was a rise in NAC species and a decline in *C. albicans* infections. Recent reports from Riyadh hospitals showed that *C. tropicalis* dominated the rise of NAC species, followed by *N. glabratus* [68, 69, 70, 71, 72]. Conversely, a study in Medina reported *C. parapsilosis* (37%) and *N. glabratus* (32%) as the main agents for the increased NAC‐related fungemia (*n* = 331) during the years 2013–2019, followed by *P. kudriavzevii, D. hansenii, C. dubliniensis*, and *Cz. auris* [65]. This shift in the species involved in yeast infections is a reflection of the rising prevalence of NAC species in Saudi Arabia. A cross‐sectional study from the Aseer region examined 143 isolates of *Candida* spp. from various sample sources (*n* = 143), including urine (72%), blood (4.2%) and cerebrospinal fluid (CSF) (0.7%). These authors reported significant increases in frequencies of *Candida* infections, including candidemia (*n* = 84), caused by *C. parapsilosis* (21.4%), *C. tropicalis* (14.3%), and *Clavispora lusitaniae* (9.5%) between 2012 and 2013 [73]. However, *C. albicans* remained the dominant species (28.6%). A 5‐year retrospective study identified five cases of IC by *C. lusitaniae* (four neonatal cases and one in an infant) out of 129 paediatric patients [70]. An 8‐year surveillance study from western Saudi Arabia (2002–2009) reported 252 cases of *Candida* bloodstream infections, with 34.1% (*n* = 86) cases of *C. albicans* and 65.9% (*n* = 166) various NAC species [74]. Malignancy was independently associated with the development of candidemia by NAC species (OR 3.24, 95% CI 1.25–8.41). Infections caused by *C. albicans* were associated with a 50% crude 12‐month mortality rate compared to 57.8% for those caused by NAC species. The study reported a high antifungal resistance rate for fluconazole with a low in vitro susceptibility of 38.5% for *C. albicans* and 52.5% for NAC [74].

Among candidemia cases (*n* = 89) reported by Al‐Tawfiq et al. (2007) [67], patients with CVC accounted for 83%, and 96% of them received broad‐spectrum antibiotics. Complex abdominal surgeries (22%), total parenteral nutrition (52%), neutropenia (9%), acute renal failure (24%), cancer (26%), and burns (15%) were additional risk factors. For all candidemia, the overall crude mortality rate was 43%. Logistic regression analysis identified two independent causes of death, namely acute renal failure (odds ratio (OR) 5.15, 95% confidence interval (CI) 1.18, 22.55, *p* = 0.03) and involvement of *C. albicans* (OR 5.91, 95% CI 1.50, 23.24, *p* = 0.01). The length of hospitalisation before the onset of candidemia was 0–270 days (mean ± SD of 29.2 ± 35.3 days) [67]. Another study reported that renal diseases were the highest risk factor (26.2%), followed by gastrointestinal diseases (20.4%), pulmonary diseases (14.02%), and cardiovascular diseases (12.5%), while cancer was the lowest (7%) [65]. Using multiple logistic regression, three factors were found to be statistically significant, namely gender, fever, and sepsis (*p*‐values 0.053, 0.017, and 0.017, respectively) [65]. The study reported that gender may influence the risk of candidemia, as females were annually more affected compared to males over the study's period. Between 2013 and 2019, the use of antifungal therapy increased by 96%, proportional to the increase in the number of fungemia episodes during this period. For both *C. albicans* and *N. glabratus* , there was a 2% rise in the resistance rate during that period [65]. Another retrospective study from a children's hospital reported a statistical analysis of risk factors related to IC and found the following values: prematurity 28.7% of patients, low birth weight in 32.6%, CVC in 45.7%, malignancy in 16.3%, immunotherapy in 15.5%, and ventilator support in 46.5% [70]. Patients with cardiac vegetation had a mortality incidence that was more than twice as high (OR: 2.9), and patients with bloodstream *Candida* infection had more than twice the mortality rate compared to those with *Candida* isolated from other sites (OR: 2.2) [70]. Patients with ventilators died at a rate of 48.3% compared to 26.1% of patients who were without such devices (*p* = 0.009), while patients in the ICU died at a rate of 43.8% compared to 24.5% of patients from other wards (*p* = 0.03). The authors also reported that patients with *C. parapsilosis* infection had the highest mortality rate (56.2%) [70].

### 3.6 UAE

In the UAE, only one single‐centre study on the demographics and clinical data of patients with candidemia was found [26] during the period 1995 to 2001. Of the 60 candidemia cases reported, 65% of patients were diagnosed with malignancy. *C. albicans* was the most common species (45%), whereas NAC represented 67% of adult patients. The second most prevalent species was *C. tropicalis* (15%), followed by *N. glabratus* (5%), *C. parapsilosis* (5%), *Polysiphonia inconspicua* (formerly known as *Candida inconspicua*) (5%), and one case each of *D. hansenii* (formerly known as *Candida famata* ) and *C. lusitaniae*. The crude mortality rate was 50%, and that attributed to candidemia was 30%. Noteworthy, the authors observed that 70% of the candidemia cases were associated with concomitant bacteremia, and most of the patients who survived were from this group [26].

## 4 Candiduria

### 4.1 Bahrain

An epidemiological study performed in two clinical centres involving 173 patients > 18 years of age conducted in 2020/2021 on candiduria among critically ill patients suffering from solid cancers or haematological malignancies yielded the following observations. The sex ratio M/F was 0.9; 85% had solid cancers and 15% had haematological malignancies. 16.8% of patients suffered from candiduria and 3.5% had candidemia. Thirty‐one urinary samples yielded *Candida* yeasts, 45% *C. albicans* isolates and the remaining various NAC species (54.8%). Of these, 42% of isolates were *C. tropicalis* , and the remaining (13%) were *N. glabratus* , *C. parapsilosis* , *Kluyveromyces marxianus* (formerly known as *Candida kefyr*) and *C. lusitaniae*. *C. albicans* and *C. tropicalis* isolates were susceptible to fluconazole. No information on susceptibility to this compound was reported for the other four isolates. Sixty‐nine patients died while undergoing treatment in the ICU. Patients with candiduria had longer hospital stays and a higher ICU morbidity when compared with non‐candiduria patients [43].

### 4.2 Kuwait

In 2002, *C. albicans* was the predominant species among *Candida* spp. isolated from urine samples in Kuwait, followed by *C. parapsilosis* , *C. tropicalis* , and *N. glabratus* [81]. In another study, urine was the source of the first cases of *C. dubliniensis* reported from Kuwait [45, 58].

In a study by Al‐Obaid et al. (2017), *C. tropicalis* isolates from 54 patients at Al‐Amiri Hospital in Kuwait, including those with candiduria, were examined. Many of the patients had urinary catheters, ureteric stents, or suprapubic catheters, and the isolates were phenotypically and genetically characterised. Most of the *C. tropicalis* isolates were susceptible to antifungal agents. Genetic analysis revealed significant diversity, with 59 diploid sequence types (DSTs), 54 of which were newly identified [47]. *C. parapsilosis sensu stricto*, *C. orthopsilosis*, *N. glabratus sensu lato*, *C. lusitaniae*, and *K. marxianus* have been detected among patients with candiduria/funguria [48, 49, 50, 51, 52, 53, 54]. Alobaid et al. (2018) [55] reported a rare case of a *Candida* fungus ball in a 37‐year‐old male patient with retroperitoneal fibrosis. Despite initial improvement with corticosteroids and bilateral percutaneous nephrostomy (PCN), the patient developed recurrent urinary tract infections (UTIs) and obstructive uropathy. The diagnosis of a fungal mass in the renal pelvis was confirmed by PCN fluid culture. PCN fluid culture grew *C. albicans* , which was susceptible to fluconazole, amphotericin B, and flucytosine. Treatment included systemic fluconazole for 2 months, local amphotericin B instillation, ureteric stenting, and laser trimming of fungal material, achieving a temporary resolution. Relapses required repeated interventions, and long‐term management involved annual stent changes and gradual withdrawal of immunosuppressive therapy [55].

### 4.3 Oman

*Candida* species were isolated in 2.3% of patients (*n* = 650) with complicated UTIs, and the presence of *Candida* underscores the need for considering fungal infections in the management of complicated UTIs [56].

### 4.4 Qatar

A 60‐year‐old diabetic man with post‐prostate surgery developed recurrent fever and signs of infection due to *C. albicans* [64]. Imaging revealed a fungal ball in the left kidney. The patient was initially treated with fluconazole, followed by anidulafungin due to persistent fever. Despite improvement in the patient's condition, urine culture remained positive for *C. albicans* . Therefore, the patient was treated with fluconazole instilled via a nephrostomy tube for 7 days, resulting in a successful outcome [64].

### 4.5 Saudi Arabia

A cross‐sectional study from the Aseer region examined 143 isolates of *Candida* in various sample sources and showed that urine was the most common source of *Candida* (72%). The authors reported an increasing frequency of infections, including candiduria, caused by *C. parapsilosis* (21.4%), *C. tropicalis* (14.3%), and *C. lusitaniae* (9.5%) between 2012 and 2013 [73]. However, specific details about the species involved in candiduria were not provided.

### 4.6 Yemen

Al‐Haifi et al. (2024) recently reported 25% cases of catheter‐associated *Candida* UTI among all patients with urinary catheters (*n* = 200) who were admitted to several hospitals of Thamar city in Yemen. *Candida* isolates were identified using conventional methods, i.e., chromogenic agar medium and germ tube test. The prevalence of *Candida* species isolated from catheter‐associated UTIs was highest among individuals aged 51–60 years (28%, *n* = 50) and lowest in those aged 10–20 years (8%, *n* = 50). Males had a higher incidence than females, comprising 28/50; 56% of cases compared to 22/50; 44% in females, and *C. albicans* (*n* = 23/50; 46%) was the most commonly identified species from these catheter‐related infections [76]. Antifungal susceptibility testing was performed using disk diffusion method based on the Clinical and Laboratory Standards Institute (CLSI) M44‐A reference method [85]. The isolates displayed the highest susceptibility to itraconazole (64%), with fluconazole and nystatin showing sensitivity rates of 60%, and 50%, respectively. Amphotericin B and ketoconazole were the least active drugs. A significant correlation was observed between catheterisation duration of < 5 days (*p* = 0.01) and antifungal susceptibility, with *Candida* isolates showing higher sensitivity to itraconazole (*p* = 0.03), ketoconazole (*p* = 0.04), and nystatin (*p* = 0.03) during this period. The authors reported that antibiotic therapy and diabetes were significant risk factors for candiduria [76].

## 5 Emergence of *Candidozyma auris* (Formerly Known as *Candida auris*)

*Candidozyma auris* has emerged as a significant global healthcare threat due to its resistance to multiple antifungal agents, high mortality rates, and persistent hospital outbreaks. Several countries have reported rising cases of *Cz. auris* infections, often linked to ICU settings, extensive antibiotic use, and comorbid conditions. Table 3 presents data on first reported cases of *Cz. auris*, isolation sources, and the number of affected patients across the region.

**TABLE 3:** First reports of *Candidozyma auris* detection across GCC countries and Yemen.

Country	Clinical source	Date of first isolation	No. of patients affected	References
Bahrain	Blood	October 2021 to November 2022	10	Alagha et al. [86]
	Urine		26	
	Soft tissue		4	
Kuwait	Blood	May 2014	1	Emara et al. [87]
Qatar	Tracheal aspirate	Dec 2018 to Aug 2019	1	Shaukat et al. [88]
	Nose		4	
	Decubitus ulcer		2	
	Throat		2	
	Groin		3	
	Sputum		1	
	Axilla		3	
	Tracheostomy site		1	
Oman	Blood	September 2016	1	Mohsin et al. [89]
		January 2017	1	
Saudi Arabia	Blood	December 2017 to February 2018	2	Abdalhamid et al. [90]
	Pleural tissue		1	
United Arab Emirates	Blood	2018	1	Alatoom et al. [75]
Yemen	No reports	No reports	NA	NA

Abbreviation: NA, not applicable.

### 5.1 Bahrain

A recent single‐centre study conducted at Salmaniya Medical Complex in Bahrain examined risk factors for 30‐day mortality in 59 patients infected or colonised with *Cz. auris*. With a mortality rate of 44.1%, the study identified significant risk factors, including age (≥ 65 years), multiple indwelling catheters, ICU admission for over 24 h, and recent intubation. *Cz. auris* was frequently isolated from various body sites, most commonly the groin (33.9%) and urine (25.4%), followed by the axillary region (23.7%), blood (10.2%), deep tracheal aspirate (5.1%), and other body fluids (1.7%). Most patients had recent antibiotic use (93.2%), and comorbidities were prevalent, including diabetes (52.5%), hypertension (67.8%), and cardiovascular disease (37.3%). While chronic kidney disease was significant in univariate analysis, it was not in multivariate analysis. The findings emphasise the need for stringent infection control and antimicrobial stewardship to reduce mortality, with early detection and a multidisciplinary approach being crucial for managing *Cz. auris* in healthcare settings [86].

### 5.2 Kuwait

The first case of candidemia caused by *Cz. auris* was diagnosed in May 2014 in a 27‐year‐old female with renal failure and admitted to the ICU [87]. The patient died the following day of diagnosis. Only sporadic cases of *Cz. auris* isolation, mostly involving urine or other yeast‐colonised body sites, were recorded in the next few months. The Mycology Reference Laboratory (MRL) re‐identified all yeast isolates that showed resistance to one or more antifungal agents, formed different shades of pink colonies on chromogenic media (CHROMagar *Candida*), or that were detected as *Cz. haemuli* by Vitek 2 YST. A *Cz. auris*‐specific PCR assay was used to screen 280 isolates, and 158 isolates from 56 patients were identified as *Cz. auris*, while only six *Cz. haemuli* and two *Candidozyma duobushaemuli* were identified [91].

Consistent with the worldwide trend, the spread of *Cz. auris* in other hospitals and healthcare facilities within Kuwait continued as the species was subsequently isolated from other facilities which were previously *Cz. auris*‐free. Despite the implementation of control measures, this rapidly increasing incidence of *Cz. auris* continued in major hospitals [45]. Surprisingly, the spread of *Cz. auris* was rapid in some hospitals while the cases remained low in other hospitals. Overall, there was a steep rise in the number of *Cz. auris*‐related candidemia cases in 2018 compared to 2017, so much so that it surpassed *N. glabratus* as the fourth most common cause of candidemia [4, 45]. A major *Cz. auris* outbreak occurred and persisted for 18 months in a secondary‐care hospital in Kuwait [3]. Twelve candidemia and 26 colonised patients were admitted or transiently exposed to the high‐dependency unit (HDU) where the outbreak started. Overall, 71 patients were affected. Despite the implementation of appropriate infection control measures, sporadic cases continued to occur even after June 2019, challenging the containment efforts. Clinical and hospital environmental isolates were identical by fingerprinting using short tandem repeat (STR) analyses [92]. Despite treatment of all candidemia and 36 *Cz. auris*‐colonised patients, 9 of 17 (52.9%) candidemia and 27 of 54 (44.4%) colonised patients died [3]. Patients with *Cz. auris* candidemia had multiple comorbidities; the duration of hospital stay before onset of candidemia varied from 5 to 93 days, and the mortality was > 50% which was consistent with the mortality rate of 0% to 72% reported previously from other countries [9, 91, 93]. Of note, *Cz. auris* was restricted to adult patients in Kuwait, contrary to observations from South Africa and some other countries [94, 95, 96]. Studies from Kuwait have also shown that multiple genotypes of *Cz. auris* with reduced echinocandin susceptibility may exist within the same patient [13, 14].

### 5.3 Oman

*Candidozyma auris* cases have been recorded from several hospitals in Oman. Almost all *Cz. auris* strains from the Middle East were resistant to fluconazole (MIC ranging from 32 to ≥ 256 mg/L) in vitro [97], although their resistance to other antifungal medications varied. Seven cases of *Cz. auris* were described in studies published in 2017 [89, 98]. Later, local data provided by Al Maani et al. (2019) [99] revealed 11 more cases. The authors examined clinical, epidemiological, and microbiological data related to an outbreak of *Cz. auris* in an Omani tertiary hospital between April 2018 and April 2019 [99]. Apart from the clinical and screening samples of the patient, environmental specimens were collected from frequently touched sites as well as from the hands of 35 staff members. MALDI‐TOF MS was used to identify all positive patient samples and environmental screening results. Of the 32 patients who were *Cz. auris* positive, 43.8% presented with UTIs, 34.4% with candidemia, and 21.8% were asymptomatic with cutaneous colonisation [99]. The median age of the patients was 64 years (range: 14–88 years), with 46.9% females and 53.1% males. Prior to diagnosis, 34.4% had been working as a nurse in a medical or surgical ward, and 65.6% were admitted to an ICU. The overall mortality rate in this patients’ cohort was 53.1%. Additionally, *Cz. auris* was isolated from two ventilator swabs of two different ICU beds. All screening samples of healthcare workers were negative. All isolates showed complete resistance to fluconazole (100%), while 33% showed resistance to amphotericin B [99]. Mohsin et al. (2020) [100] examined retrospective data from 2016 to 2018 on the epidemiology, clinical presentation, and microbiology of 23 episodes of *Cz. auris* fungemia at Oman's Royal Hospital, a tertiary‐care facility. MALDI‐TOF MS was used to identify the yeast isolates. It was found that *Cz. auris* was the cause of fungemia in 23 individuals. According to the results of microsatellite genotyping, the isolates from Oman were part of the South Asian clade I [100]. All isolates were resistant to fluconazole and susceptible to echinocandins; hence, the primary course of treatment was initiated with echinocandins. Eatemadi et al. (2019) [101] reviewed all positive blood culture reports that showed positive *Cz. auris* at Suhar Teaching Hospital between May 2018 and the end of April 2019. At Oman's Central Public Health Laboratory (CPHL), the isolates were identified using MALDI‐TOF MS, and Vitek 2 YST was used to perform antifungal susceptibility testing. Eight out of 13 individuals with candidemia had medical comorbidities, prolonged hospital stays, or had been exposed to antibiotics for an extended period. The time gap between being admitted to the hospital and the onset of *Cz. auris* candidemia varied from 8 to 49 days, with a median of approximately 27 days. Resistance to amphotericin B and fluconazole was observed in isolates from five patients, and all isolates exhibited moderate sensitivity to voriconazole and high sensitivity to echinocandins, i.e., caspofungin and anidulafungin. Four patients were treated with fluconazole (3/4 died), eight patients received echinocandins (4/8 died), and one patient was discharged without therapy. The 30‐day mortality rate was 61.5% overall.

Al‐Rashdi et al. (2021) [102] conducted another retrospective analysis of *Cz. auris* infections that were reported to the Oman Antimicrobial Resistance Surveillance System countrywide in 2019. The findings demonstrated that *Cz. auris* infections persisted and posed a serious threat to public health. Clinical samples from 108 inpatients yielded 129 *Cz. auris* isolates. On average, 3.5 months passed between the patients' admission and infection, during which 52.5% of them died, even though the isolates were susceptible to echinocandins in vitro [102]. Amphotericin B and fluconazole had no effect on 94.8% and 96.1% of the isolates, respectively [102]. An average of 3.5 months passed between the patients' admission and infection, during which 52.5% of them died, even though the isolates were susceptible to echinocandins in vitro [102]. A retrospective descriptive cohort study at Royal Hospital was conducted on adult inpatients between 2017 and 2020 [103]. During the study period, any patient who tested positive for *Cz. auris* in a screening or clinical sample was considered to have either an infection or a colonisation. Since 2017, the overall incidence of *Cz. auris* increased, and this was attributed to several factors, such as the COVID‐19 pandemic and the implementation of active surveillance. Two recent hospital admissions over the last 6 months and diabetes mellitus were statistically significant risk factors associated with *Cz. auris* infection. The mortality rate was 40.0%, and the hospital stay duration median was 31 (1–380) days. Age, chronic kidney disease, intubation, and infection were statistically significant risk factors for mortality [103]. Two other Omani cases of *Cz. auris* were reported separately; the first included a 50‐year‐old diabetic male with sickle cell disease, intact neurology, and a history of multiple surgeries [104]. *Cz. auris* was isolated from the infected central line and identified using MALDI‐TOF MS. The patient was treated with caspofungin [104]. The second case involved a 56‐year‐old male who was admitted with H1N1‐related pneumonia. The patient was intubated and subsequently developed a bloodstream infection caused by *Cz. auris*, likely due to extended antibiotic use and an immunocompromised state [105]. The patient was successfully treated with anidulafungin.

### 5.4 Qatar

Shaukat et al. (2020) described the clinical manifestations and outcomes of *Cz. auris* infections for inpatients of a general hospital in Qatar from December 2018 to August 2019 [88]. The study included 13 patients with five invasive *Cz. auris* infections and eight with superficial colonisation. Among patients who were clinically infected with *Cz. auris*, two suffered from UTIs, one was diagnosed with candidemia, another patient had a soft tissue infection, and one presented with a lower respiratory tract infection. All strains of *Cz. auris* were susceptible to echinocandins, flucytosine, and posaconazole but resistant to fluconazole and amphotericin B. Among the patients with *Cz. auris* infection who were treated with systemic antifungal therapy (*n* = 5), 60% died during the course of treatment [88].

In the context of the COVID‐19 pandemic, several studies explored the incidence of candidemia and drug resistance, particularly *Cz. auris*, which was shown to be prevalent in ICU patients and exhibited high resistance rates to antifungal drugs. Goravey et al. (2021) [106] documented *C*z. *auris* fungemia in a 64‐year‐old man suffering from acute respiratory distress syndrome (ARDS) induced by severe COVID‐19. The patient developed ventilator‐associated pneumonia and sepsis with various bacterial and fungal infections that were resistant to common antimicrobial drugs. Despite extensive interventions like extracorporeal membrane oxygenation (ECMO) and haemodialysis, the patient deteriorated and passed away. The authors emphasised that both ARDS and ECMO posed higher risks of secondary and opportunistic infections such as invasive fungal disease [106].

The genomic epidemiology and antifungal resistance of *Cz*. *auris* in Qatar were investigated by Salah et al. (2021) [107]. The authors examined the whole genomes of 44 *Cz. auris* isolates (40 clinical and four environmental isolates) collected from April 2018 to November 2020 from three major hospitals. Three isolates were obtained from invasive infections, whereas 37 isolates were considered as colonisers. The genomic analysis showed that all isolates were of South Asian origin and showed low genetic variability of isolates originating from two hospitals, whereas three isolates from one patient in the third hospital clustered separately. The four environmental isolates were collected from the same hospital, with one found on a bedside table and three others on the bed, couch, and cabinet in a different patient's room. These environmental isolates were genetically identical to those from clinical samples. One patient was previously hospitalised in Oman, while another received medical care in Sudan and India. The findings emphasised the potential role of international travel in the transmission of *Cz. auris* across countries.

Between March 2020 and June 2021, Ben Abid et al. (2023) further explored *Cz. auris* isolates and drug resistance mechanisms during Qatar's COVID‐19 pandemic. Examining genomes of 76 isolates, of which 65 were obtained from patients with COVID‐19, they found that over 70% of the isolates were resistant to fluconazole and amphotericin B based on antifungal susceptibility testing [108]. Genomic analysis identified resistance‐associated mutations linked to azole and echinocandin resistance, in addition to a novel mutation that might be associated with amphotericin B resistance. The isolates were isolated from screening samples (*n* = 42), blood (*n* = 1), urine (*n* = 13), respiratory samples (*n* = 4), and wound specimens (*n* = 3). Low genetic variability was observed among the isolates from patients with or without COVID‐19, confirming an outbreak with genetically identical strains and the ongoing dissemination of *Cz. auris* among various healthcare facilities within Qatar.

Garcell et al. (2023) [109] investigated the incidence of health care associated‐infections in a medical‐surgical ICU in western Qatar during 10 years and compared the incidence during the pre‐COVID‐19 and COVID‐19 periods. The authors reported a higher frequency of *Candida* infections during the COVID‐19 pandemic period, especially cases of central line‐associated bloodstream infection (CLABSI) and *Cz*. *auris*. Most patients were previously colonised with *Cz. auris* [109]. Additionally, Koleri et al. (2023) [110] analysed 36 cases of *Cz. auris* fungemia in Qatar, mostly in ICU patients recovering from severe COVID‐19. The 30‐day mortality rate was 41.6%, with over 90% of isolates resistant to fluconazole and 85% resistant to amphotericin B.

Recently, Al Ajmi et al. (2024) [111] investigated the spectrum of infection and risk factors for invasive *Cz. auris* infections in Qatar, involving 331 patients with positive *Cz. auris* cultures between May 2019 and December 2022. The median patient age was 56 years, with the majority being male (83.1%). Colonisation occurred in 86.4% of cases, while 13.6% developed invasive infections. Risk factors for invasive *Cz. auris* infection included invasive central venous or urinary catheterisation and mechanical ventilation, with those affected experiencing longer ICU stays (26 vs. 16 days), hospitalisations (63 vs. 43 days), and significantly higher 30‐day mortality (38% vs. 14%). Mechanical ventilation was the strongest predictor of invasive disease (OR 3.33) [111].

### 5.5 Saudi Arabia

Between 2019 and 2023, the General Directorate of Infection Prevention and Control (GDIPC) registered cases of *Cz. auris* reported in governmental hospitals. The highest record was in the Central and Western regions of the country, with 379 and 169 cases, respectively, followed by the Eastern, Southern, and Northern regions with 70, 23, and 18 cases reported, respectively. Other non‐ and semi‐governmental centres have further characterised and sequenced *Cz. auris* isolates. As a result, the GDIPC established a national *Cz. auris* prevention strategy (NCAPS) led by a dedicated project team. This team is responsible for executing surveillance programmes to track trends and identify high‐risk areas, implement infection control measures within healthcare settings to minimise transmission, and conduct research on new diagnostics, antifungal drugs, and preventive strategies. An essential objective of the NCAPS is also to increase awareness among healthcare professionals and the general public regarding risk factors and preventive measures [112].

In 2018, the first cases were reported from three female patients admitted to the adult ICU of two hospitals in Dammam and Riyadh [90]. The patients had no history of travel in the 6 months prior to the infection. A number of comorbid conditions, such as lupus nephritis, diabetes mellitus, hypertension, severe pancreatitis, chronic heart failure, and empyema, were recorded. Other risk factors included receiving antibiotic treatment, surgical operations, and placement of CVC. Although the patients did not have a bloodstream infection, a 30‐day increase in mortality was observed in the infected patients compared with those who were colonised [90]. Fluconazole resistance was detected in seven out of the eight isolates, while amphotericin B resistance was present in five of them. Based on whole genome sequencing (WGS), all isolates belonged to the South Asian clade with two separate subclades, indicating two separate introductions and continuous transmission within facilities [113]. Between 2018 and 2019, an outbreak investigation in a tertiary‐care unit in Riyadh revealed a total of 23 primary positive patients and 11 postexposure patients' cases [114]. The cases were identified in ICUs (51.4%) and wards (48.6%). There were more cases of infection (62.9%) than colonisation (37.1%). The two most common illnesses were candidemia (17.1%) and UTIs (42.9%) [114]. Six (17.1%) of the seven patients (20.0%) who passed away while in the hospital did so within 30 days after diagnosis [114]. Another two cases were positive for *Cz. auris* in patients with lengthy hospital stays and repeated admissions to ICU due to polymorbidity [115]. In both cases, MALDI‐TOF MS verified the urine cultures isolates as *Cz. auris* . Caspofungin was prescribed for both patients, but they unfortunately died. Between the years 2015–2022, a university hospital in Jeddah reported 27 patients, of which 14 had invasive *Cz. auris* infection in a retrospective analysis [116]. The study revealed risk factors, such as the presence of an implanted device (100%), UTIs (14.8%), CLABSI (63%), fever and shock (33.3%), admission to the ICU (81%), diabetes and heart disease (48.1%), and 66.7% of the patients were males [116].

### 5.6 UAE

In 2018, the first case of *Cz. auris* from the UAE was reported from a female patient with multiple comorbidities that resulted in a protracted hospital stay with persistent candidemia. The patient passed away 3 months after the first isolation of *Cz. auris* from her blood culture [75]. Extrapolating from the National Antimicrobial Resistance surveillance database, Thomsen et al. (2023) [93] reported a total of 908 non‐duplicate *Cz. auris* isolates from 2018 to 2021 (2018: *n* = 9; 2019: *n* = 93; 2020: *n* = 192; 2021: *n* = 614). The majority of *Cz. auris* isolates were predominantly isolated from urine samples (30.8%), followed by blood (27.3%), skin and soft tissue samples (24.3%), the respiratory tract (15.6%), genital tract (0.3%), and CSF specimens (0.2%). A highly skewed distribution was found across inpatient settings in the demographic data linked to *Cz. auris* isolation (89%); most of them were ICU patients (45.6%). Male patients in the adult age group made up most of the patient population. Data on the nationality of patients was only available for 632 patients; 29.4% of them were UAE nationals, and the remainder (70.6%) originated from 34 other countries [93]. A statistically significant difference was noted when comparing ICU admissions between patients with *Cz. auris* versus other yeast infections. Of the 19,353 patients associated with *Candida* spp. (non‐*auris*), 20.2% were admitted to ICUs, as opposed to 49.6% of patients with a *Cz. auris* infection (*n* = 835). The median length of hospitalisation (length of stay, LOS) also differed significantly between both groups; patients infected with *Cz. auris* (*n* = 140) had a median LOS of 33.5 days compared to 14.0 days for patients associated with *Candida* spp. (i.e., non‐*auris*). Furthermore, Thomsen et al. (2023) reported a mortality rate of 27.5% among patients with *Cz. auris* (47/171 patients from all sources) whose admission and discharge dates were available. The mortality rate for patients associated with *Cz. auris* candidemia was 36.1% (*n* = 61 patients) [93].

### 5.7 Yemen

Although *Cz. auris* has emerged globally, there have been no reported cases from Yemen.

## 6 Discussion

The incidence of invasive and non‐invasive fungal infections has steadily increased worldwide during the last few decades, particularly among immunocompromised patient populations. *Candida* and other yeast infections represent a major component of invasive and non‐invasive mycoses globally, including the countries of the Arabian Peninsula in the Middle East [1, 2, 3, 4, 5]. Global guidelines for the diagnosis and management of candidiasis have also been recently published [117]. In this review, we have presented epidemiological features and trends, clinical manifestations, species distribution, and outcomes available for candidemia, candiduria, and *Cz. auris* infections in GCC countries and Yemen on the Arabian Peninsula.

The overall incidence of candidemia varied throughout this region. *Candida* species prevail, with *C. albicans* being the most frequently isolated organism in all countries but varying in prevalence, and a gradual shift to NAC species has been recently noted in various countries [26, 42, 44, 62, 65, 118]. This shift is clinically important because NAC species, such as *N. glabratus* , *Cz. auris*, *Cz. haemuli*, and *P. kudriavzevii*, are often less susceptible to common antifungal drugs like fluconazole, leading to treatment challenges and potential antifungal resistance [119].

Like global trends, *Cz. auris* also poses serious health risks in several countries across the region, including those with appropriate diagnostic capacity and awareness. Several outbreaks of *Cz. auris* invasive/non‐invasive infections have been described in Kuwait, Qatar, Oman, Saudi Arabia, and the UAE in recent years, which have been associated with high mortality rates.

Despite the valuable insights provided by this review, several gaps in knowledge remain. Data on *Candida* infections from some countries, such as Bahrain and Yemen, are limited, hindering a comprehensive understanding of the regional epidemiology. Additionally, the lack of standardised surveillance systems and antifungal susceptibility testing in many healthcare facilities contributes to underreporting and misdiagnosis.

A major goal of this collaborative work is also to set up a regional system to share information on the clinical, epidemiological, and molecular characteristics of *Cz. auris*‐related infections and share experiences on best management and infection control strategies in the region.

## 7 Conclusions and Recommendations

The state of the art with respect to knowledge on the epidemiology of candidemia and candiduria varies greatly between GCC countries and Yemen within the Arabian Peninsula. The rise of NAC species, including *Cz. auris*, as observed in all countries for which data are available, is alarming. Increasing awareness of the occurrence of clinically emerging rare yeast species that might be resistant to the commonly used antifungal drugs is important.

We hope that this review will stimulate local and regional epidemiological studies that will generate more insight and a better understanding of the prevalence and trends of these infections. Such studies are crucial to guiding healthcare authorities in improving patient management and optimising antifungal treatment strategies. Because of the differences in medical mycology diagnostic capacity, availability of expert mycologists, and research interests between the various countries, the amount and quality of data related to *Candida* infections vary accordingly.

To improve diagnostic capacity in the region, we suggest the following:

- A national registry for all fungal isolates and resistance patterns, which can later be integrated as a GCC‐wide registry; starting with the clinically most relevant species such as *Cz. auris*.
- Establish GCC guidelines/recommendations on infection control measures and the management of nosocomial *Candida* outbreaks, especially *Cz. auris*. Increasing awareness among healthcare professionals and the public regarding the occurrence of clinically emerging rare yeast species.
- Enhance collaboration between countries to realise unified surveillance studies and studies on the efficacy of new antifungal drugs in the region
- Proper laboratory diagnosis is not available in all countries or regions, so diagnostic capacity should be expanded
- A GCC initiative for training and education on fungal infections and their clinical implications, including the organisation of regional congresses, workshops, and courses in medical mycology, should be actively pursued.
- Encourage collaboration and information exchange on the epidemiology of yeast infections between countries in the Arabian Peninsula as well as other regional and international mycological societies for a more comprehensive understanding of the regional landscape.
- Formation of a regional medical mycological society with representation in each country to enhance collaboration and sharing of knowledge and expertise
- Increase awareness among healthcare professionals and the public regarding the risks associated with emerging fungal pathogens. This includes education on the importance of early diagnosis, appropriate treatment, and infection prevention practices.

## Author Contributions

**Suhail Ahmad:** conceptualization, methodology, data curation, writing – review and editing, writing – original draft, formal analysis. **Teun Boekhout:** supervision, methodology, conceptualization, writing – review and editing, data curation, writing – original draft, formal analysis. **Abdullah M. S. Al‐Hatmi:** writing – review and editing, data curation. **Ahmed Al‐Harrasi:** writing – review and editing, data curation. **Aiah Mustafa Khateb:** data curation, writing – review and editing. **Fatima Al Dhaheri:** data curation, writing – review and editing. **Hajer Bin Shuraym:** data curation, writing – review and editing. **Jens Thomsen:** data curation, writing – review and editing. **Khaled Alobaid:** data curation, writing – review and editing. **Mohammad Asadzadeh:** data curation, writing – review and editing. **Saad J. Taj‐Aldeen:** data curation, writing – review and editing. **Saleh Alwasel:** data curation, writing – review and editing. **Wadha Alfouzan:** data curation, writing – review and editing. **Ziauddin Khan:** data curation, writing – review and editing. **Husam Salah:** data curation, supervision, writing – review and editing, methodology, conceptualization, funding acquisition, formal analysis, writing – original draft.

## Ethics Statement

The authors have nothing to report.

## Conflicts of Interest

The authors declare no conflicts of interest.

## Data Availability

The authors have nothing to report.
